# Proteomic changes of aryl hydrocarbon receptor (*AhR*)-silenced porcine granulosa cells exposed to 2,3,7,8-tetrachlorodibenzo-*p*-dioxin (TCDD)

**DOI:** 10.1371/journal.pone.0223420

**Published:** 2019-10-04

**Authors:** Karina Orlowska, Sylwia Swigonska, Agnieszka Sadowska, Monika Ruszkowska, Anna Nynca, Tomasz Molcan, Agata Zmijewska, Renata E. Ciereszko

**Affiliations:** 1 Department of Animal Anatomy and Physiology, Faculty of Biology and Biotechnology, University of Warmia and Mazury in Olsztyn, Oczapowskiego, Olsztyn, Poland; 2 Laboratory of Molecular Diagnostics, Faculty of Biology and Biotechnology, University of Warmia and Mazury in Olsztyn, Prawochenskiego, Olsztyn, Poland; Temple University, UNITED STATES

## Abstract

2,3,7,8-tetrachlorodibenzo-*p*-dioxin (TCDD) is a toxic man-made chemical compound contaminating the environment and affecting human/animal health and reproduction. Intracellular TCDD action usually involves the activation of aryl hydrocarbon receptor (AhR). The aim of the current study was to examine TCDD-induced changes in the proteome of *AhR*-silenced porcine granulosa cells. The *AhR*-silenced cells were treated with TCDD (100 nM) for 3, 12 or 24 h. Total protein was isolated, labeled with cyanines and next, the samples were separated by isoelectric focusing and SDS-PAGE. Proteins of interest were identified by MALDI-TOF/TOF mass spectrometry (MS) analysis and confirmed by western blotting and fluorescence immunocytochemistry. The *AhR*-targeted siRNA transfection reduced the granulosal expression level of *AhR* by 60–70%. In *AhR*-silenced porcine granulosa cells, TCDD influenced the abundance of only three proteins: annexin V, protein disulfide isomerase and ATP synthase subunit beta. The obtained results revealed the ability of TCDD to alter protein abundance in an AhR-independent manner. This study offers a new insight into the mechanism of TCDD action and provide directions for future functional studies focused on molecular effects exerted by TCDD.

## Introduction

Polychlorinated dibenzo-*p*-dioxins (PCDDs) are widely distributed and highly toxic environmental contaminants produced by various industrial processes (e.g., metal smelting processes, paper pulp bleaching). Dioxins may also be introduced to the environment by incineration of hospital and industrial waste, burning in house furnaces, car traffic and cigarette smoking. The most toxic PCDD congener is 2,3,7,8-tetrachlorodibenzo-*p*-dioxin (TCDD). Because of its lipid solubility and high chemical stability as well as resistance to biodegradation, TCDD easily accumulates in human and animal tissues [1, 2]. In humans, the half-life of TCDD is estimated to be 7–11 years, while in the environment it ranges from 25 to 100 years [3].

The effects of dioxins are mediated mainly *via* the activation of aryl hydrocarbon receptor (AhR). AhR is a ligand-activated transcription factor belonging to the basic-helix-loop-helix (bHLH) Per-Arnt-Sim (PAS) family. After exposure to TCDD, the TCDD/AhR complex translocates to the nucleus where it couples with AhR nuclear translocator (ARNT). The TCDD/AhR/ARNT complex binds to a specific xenobiotic responsive element (XRE) located upstream in the promoter region of the target genes [4]. Other signaling pathways triggered by TCDD have been discussed, but are still under investigation [5, 6]. Although it is commonly believed that xenoestrogens may also act *via* the genomic activation of other intracellular receptors such as pregnane X (PXR) and constitutive androstane (CAR) receptors [7], there is no *in vitro* evidence indicating their involvement in TCDD signaling pathway. However, it was demonstrated that estrogen [8] and androgen [9] receptors may be involved in TCDD signaling pathway. In addition, *in silico* studies demonstrated that TCDD is able to bind with PXR and CAR with high binding affinity [10]. Similar observations were recorded for glucocorticoid, progesterone and mineralocorticoid receptors [11]. TCDD, similar to other xenoestrogens, also activates some non-genomic pathways involving e.g., tyrosine (Src) kinases, protein kinase A, protein kinase C, cAMP, nitric oxide and calcium ions [12, 13]. The presence of AhR-independent TCDD signaling pathways in granulosa cells has yet to be elucidated.

Exposure to TCDD may result in a variety of harmful short- and long-term effects, such as wasting syndrome, cancer and neurological dysfunctions. TCDD has also been demonstrated to cause reproductive defects and endocrine disruption in porcine ovaries [14, 15, 16]. Granulosa cells play a fundamental role in the proper growth, development and functioning of ovarian follicles [17]. They produce steroid hormones to support oocyte maturation and to ensure an optimal environment for fertilization, implantation and embryo development. Disruption of granulosal steroidogenesis may lead to follicular dysfunction and atresia as well as may affect functions of the entire female reproductive tract.

Due to the fact that TCDD was found to affect progesterone and estradiol production by granulosa cells in pigs [14, 15, 18] it is important to indicate molecular targets of TCDD in these cells. The results of our previous studies demonstrated that TCDD affected the expression of genes involved in cell cycle, proliferation and follicular atresia [19] as well as the expression of long non-coding RNAs (lncRNAs) [20]. It was also demonstrated that TCDD may affect the ovarian follicle fate by the rearrangement of the cytoskeleton and the extracellular matrix (ECM) as well as the modulation of proteins important for cellular response to stress [21]. All these studies were performed on porcine granulosa cells reported to exhibit AhR expression [22]. In contrast, the goal of the current study was to examine whether TCDD may affect the proteome of porcine granulosa cells in a way different to the canonical AhR-mediated pathway and to identify molecular components of such pathways. In the present study we aimed, for the first time, to identify proteins involved in the mechanism of TCDD action in *AhR*-silenced porcine granulosa cells. To meet this goal we applied RNA interference (RNAi) technology, two-dimensional fluorescence difference gel electrophoresis (2D-DIGE) and mass spectrometry (MS). The obtained results, together with those reported previously, allow for the complex exploration of TCDD-induced changes in the porcine ovary.

## Materials and methods

### Culture of porcine granulosa cells

All experiments were carried out on AVG-16 cell line (The European Collection of Authenticated Cell Cultures; 06062701; Salisbury; UK) derived from porcine granulosa cells of medium follicles [22, 23]. Before the experiment, the cells were thawed and cultured as previously described [19, 21, 22]. After reaching 60–70% confluency, the cells were washed twice with sterile phosphate-buffered saline (PBS), and fresh culture medium was added. The cells were then used either as control untransfected cells (UTR) or were transfected with small interfering RNAs (siRNAs).

### AhR gene silencing in porcine granulosa cells

Porcine granulosa cells were transfected with siRNA using Viromer^®^ BLUE (Lipocalyx GmbH, Halle, Germany). Three different siRNAs targeting mRNA of AhR (anti-*AhR* 1; anti *AhR* 2; anti-*AhR* 3; Table 1) were synthesized by Sigma Aldrich. As a negative control, siRNA duplex with an irrelevant sequence (Thermo, Waltham, MA, USA) was applied. Each of the lyophilized siRNAs were dissolved in RNase-free water, producing a 20 μM stock solution. Next, each of the three siRNAs was diluted with Buffer Blue to a treatment concentration of 2.8 μM and all the siRNAs were pooled at equimolar concentration to improve the gene silencing efficiency. The transfection reagent—Viromer^®^ BLUE—was diluted 90× in Buffer Blue and combined with the siRNA mixture. This step was followed by the 15 min incubation (room temperature). Finally, the transfection mixture (3 ml) was added to the cells in a drop-wise manner and was incubated for 24 h at 37°C in a 5% CO_2_ humidified atmosphere. The cells with a fully active AhR gene (i. e., untransfected cells, UTR) were employed as control cells. To confirm the silencing of AhR gene in porcine granulosa cells, the expression of *AhR* was determined in: 1/ UTR cells, 2/ cells transfected with irrelevant siRNA sequence (TR_NEG_) and 3/ cells transfected with the three relevant siRNAs (TR) by quantitative real-time polymerase chain reaction (qRT-PCR) using gene-specific primers (Table 2).

**Table 1:** The sequences of siRNAs used to silence AhR gene in porcine granulosa cells.

Target Gene	siRNA name	Sense/Antisense strand	siRNA sequence (5’→3’)
AhR	anti-*AhR* 1	Sense	GCAAGAUGAGUCUGUUUAUdTdT
		Antisense	AUAAACAGACUCAUCUUGCdTdT
	anti-*AhR* 2	Sense	CUUUACACCUACUGGUUGUdTdT
		Antisense	ACAACCAGUAGGUGUAAAGdTdT
	anti-*AhR* 3	Sense	GCUGUUCUCUAUGAGAUAAdTdT
		Antisense	UUAUCUCAUAGAGAACAGCdTdT

AhR–aryl hydrocarbon receptor

**Table 2:** Primers and probes used for qRT- PCR.

Gene symbol	Sense/Antisense strand	Primer sequences (5’→3’)	Probe sequences
AhR	Sense	TGGAAGACCAGATTATATCATTGCAACTC	TTCATCTGTGAGAGGTCTCT
	Antisense	GCGTTTTCGTAGATGTTCTTTTCCT	
β-actin	Sense	GCTCTTCCAGCCCTCCTT	CTGGGCATGGAGTCCT
	Antisense	GTTGAAGGTGGTCTCGTGGAT	
GAPDH	Ss03373286_u1, Thermofisher Scientific, Waltham, MA, USA		

AhR—aryl hydrocarbon receptor. β-actin and glyceraldehyde 3-phosphate dehydrogenase (GAPDH)—reference genes

### TCDD treatment of the cells

In the current study, we compared the proteomes of *AhR*-silenced porcine granulosa cells (TR) incubated in the presence or absence of TCDD. Twenty four hours after the transfection of granulosa cells, culture medium was exchanged, and the TR cells were incubated for 3, 12 or 24 h (n = 4/time point) in the absence (control TCDD-untreated TR cells) or in the presence of TCDD (100 nM, TCDD-treated TR cells; Sigma Aldrich). The TCDD concentration was selected on the basis of previously published data [24, 25], where 100 nM of TCDD was found to affect granulosa cell steroidogenesis of pigs but did not affect the cell viability [25]. Although 100 nM is rather not considered to be an environmentally relevant dose, nanomolar concentrations of TCDD may be occasionally found in living organisms [26]. Moreover, since the goal of the current experiment was to reveal as many as possible molecules (pathways) involved in the cellular mechanism of TCDD action, the supraphysiological TCDD concentration, without viability effects seemed to meet our expectations. After incubation, medium was removed, the cells were washed twice with PBS and then total RNA and protein were isolated.

### Total RNA isolation and qRT- PCR

Total RNA was isolated from cells using peqGold TriFast (Peqlab Biotechnologie GmbH, Erlangen, Germany). RNA concentration and quality were determined using NanoVue Plus spectrophotometer (NanoVue Plus, GE Healthcare, Little Chalfont, UK). To test the extent of the silencing of AhR gene in porcine granulosa cells after siRNA transfection, qRT-PCR was performed. Complementary DNA was generated from total RNA isolated from four biological replicates of UTR (n = 4), TR_NEG_ (n = 4) and TR (n = 4) granulosa cells per each time point using the Omniscript RT Kit (Qiagen, Hilden, Germany) with 0.5 μM oligo(dT)_15_ primer (Roche, Basel, Switzerland), 1 μM hexanucleotide primers and 10 U RNase Out (Sigma Aldrich) in a Veriti Thermal Cycler (Thermofisher Scientific, Waltham, MA, USA) at 37°C for 1 h. Specific primers and probes for particular genes were synthesized by Thermofisher Scientific Company (Table 2). Glyceraldehyde 3-phosphate dehydrogenase (GAPDH) and β-actin were used as reference genes. qRT-PCR was performed using TaqMan® Universal PCR Master Mix and TaqMan Gene Expression Assay (Thermofisher Scientific) in Applied Biosystems 7500 Fast Real-Time PCR System (Thermofisher Scientific). The amplification cycle was as follows: initial denaturation at 95°C for 10 min, 40 cycles of denaturation at 95°C for 15 s and primer annealing at 60°C for 1 min. The qRT-PCR for each of four biological replicates was carried out in duplicate, and non-template control was included in each run. Gene expression levels were normalized to GAPDH and β-actin to attain the relative expression by using comparative cycle threshold (C_T_) method and quantity based active schematic estimating (Q-BASE) model, and they were expressed as arbitrary units (mean ± SEM) [27]. The differences in AhR gene expression levels between UTR, TR_NEG_ and TR granulosa cells were evaluated using one-way ANOVA (Statistica Software Inc., Tulsa, OH, USA). Differences with a probability of p<0.05 were considered significant.

### Protein isolation

Proteins were extracted with lysis buffer (7 M urea, 2% *w*/*v* CHAPS, 2% ampholytes [pH 4–7 NL; GE Healthcare, Chicago, IL, USA], 120 mM dithiothreitol, protease inhibitors cocktail [Sigma Aldrich], 0.002% bromophenol blue). The isolation and purification procedures were previously described in detail [21]. The protein concentration was determined before and after purification, using 2D-PAGE adapted Bradford assay [28] with BSA dissolved in rehydration buffer (7 M urea, 2 M thiourea, 2% CHAPS, 130 mM DTT, 2% ampholytes [pH 4–7 NL]) as a protein standard. BSA dilutions and the examined samples were acidified with 10 μl of 0.1 M HCl. The measurements were carried out at a wavelength of 595 nm using the Infinite M200 PRO multimode microplate reader (Tecan, Männedorf, Switzerland). The isolation procedure allowed for obtaining an average of 3 500 μg of proteins per 10^6^ cells (ranging from 2 700 to 3 900 μg of proteins). The mean yield of subsequent protein purification was 85% (65–91%). The obtained protein extracts were used in 2D-DIGE and Western blotting.

### Protein sample labeling and 2D-DIGE

Aliquots of 50 μg of protein from each sample (control and TCDD-treated TR cells, *n* = 4/ treatment/ time point) were dissolved in labeling buffer (30 mM Tris, 7 M urea, 2 M thiourea, 4% *w*/*v* CHAPS, pH 8.0) and labelled with CyDye DIGE Fluor minimal dyes (GE Healthcare, reconstituted in fresh 99.8% anhydrous dimethylformamide) at concentration of 400 pmol dye/50 μg of protein. A dye swap (Cy3/Cy5) of control and TCDD-treated samples was performed to exclude dye bias. An internal standard was created by mixing equal amounts of each of 24 experimental samples listed in S1 Table. The protein aliquots of internal standard as well as aliquots of control and TCDD-treated samples were labeled with Cy2, Cy3 or Cy5 according to the pattern presented in S1 Table, which was followed by incubation on ice for 30 min in the dark. The gel-loading mixtures (n = 12; S1 Table) were enriched with a rehydration solution (7 M urea, 2 M thiourea, 2% CHAPS, 2% pharmalyte pH 4–7, 130 mM DTT and a trace of bromophenol blue) to a final volume of 450 μl. The mixtures were loaded onto 24 cm immobilized pH gradient strips (pH 4–7, GE Healthcare). The isoelectricfocusing, protein equilibration and second dimension (SDS-PAGE) were performed as previously described [21].

### Image acquisition and analysis

Individual gels were scanned using Ettan DIGE Imager (GE Healthcare) to visualize the spots. Image analysis was performed with SameSpots (Totallab, Newcastle, UK). The obtained volume of each spot was normalized against the volume of the Cy2 labeled internal standard spot. In order to investigate the TCDD-induced proteome changes, the spots derived from control samples and TCDD-treated samples were matched. The spots with significant (p<0.05 and fold change≥1.5) abundance changes between control and TCDD-treated samples (differentially expressed protein spots; DEPSs) were designated to mass spectrometry for protein identification.

### Protein digestion and MALDI-TOF/TOF analysis

To properly pick and identify the selected spots, DIGE gels were restained using Coomassie Brilliant Blue G-250 (BioRad). The protein digestion and MALDI-TOF/TOF MS analysis were performed as previously described [21]. Statistical probability of the correct prediction of the identified protein was calculated by the MASCOT, including peptide mass fingerprint and ion scores. Scores above 70 (p<0.05) were considered significant.

### Western blotting

Western blotting was used to confirm the abundance of three identified proteins (annexin V [AnxA5], protein disulfide isomerase [PDI] and ATP synthase subunit beta [ATPβ]) determined by 2D-DIGE and MS. An aliquot of 10–20 μg of each individual sample protein was resolved by SDS-PAGE (12.5% polyacrylamide gel) and transferred to nitrocellulose membranes (GE Healthcare) as previously described [21]. Then, the membranes were incubated (3 h, with shaking) with rabbit polyclonal antibodies (Abcam, Cambridge, UK; diluted in TBST, 1:500 for AnxA5, 1:1 000 for PDI and 1:500 for ATPβ) or with goat polyclonal antibodies (Abcam, Cambridge, UK; diluted in TBST, 1:500 for reference protein–β-actin). After incubation with the primary antibodies, the membranes were washed three times with TBST and incubated for 1 h at room temperature with HRP-conjugated goat anti-rabbit IgG (Abcam; diluted in TBST, 1:5 000) or donkey anti-goat secondary antibodies (Abcam; diluted in TBST, 1:10 000). Immunolabelled bands were visualized using Immobilon chemiluminescent HRP substrate (Millipore, 205 Billerica, MA, USA) according to manufacturer instructions. The results of the western blot were quantified by densitometric scanning of immunoblots (n = 3 replicates for each protein) with Image Studio Lite (version 5.2). The densitometric analysis of the selected proteins was performed in relation to a reference protein (β-actin), providing data presented as arbitrary optical density units. The arbitrary units were expressed as percentages of corresponding control samples (100%). The raw data were analyzed by Student's t-test (p<0.05).

### Immunofluorescence staining

The effects of TCDD on changes in AnxA5 and PDI immunofluorescence staining were measured in the *AhR*-silenced porcine granulosa cells (TR) cultured (LabTek Chamber Slide Systems, Nunc, Denmark) with TCDD (100 nM) for 12 h (n = 3 independent experiments). Following the culture, the cells were fixed in 4% paraformaldehyde and incubated (12 h) with primary rabbit polyclonal antibodies against AnxA5 (1:500) or PDI (1:300). The primary antibodies were omitted in negative control samples to confirm the specificity of the assay. Next, the cells were incubated (1 h) with goat anti-rabbit biotinylated secondary antibodies (1:100), and then treated with fluorescein isothiocyanate (FITC, green stain) conjugated with streptavidin (1:50). To visualize cellular nuclei, the cells were stained with propidium iodide (red stain). Fluorescence intensity of stained cells was determined using the NIS-Elements 3.0 Imaging System (Nikon, Tokyo, Japan). In addition to acquiring cell images, the optical density of fluorescence staining was also measured. To ensure the objectivity of the procedure, six images were taken consistently from the same six precisely defined areas of each well. Each cell was selected as a region of interest (ROI) and the mean fluorescence intensity of all selected cells present in the image was calculated. Data were expressed as arbitrary units representing the intensity of granulosa cell staining. The raw data were analyzed by Student's t-test (p<0.05).

## Results

### The effect of the AhR-targeted siRNA transfection on the AhR expression level in porcine granulosa cells

**Fig 1:**
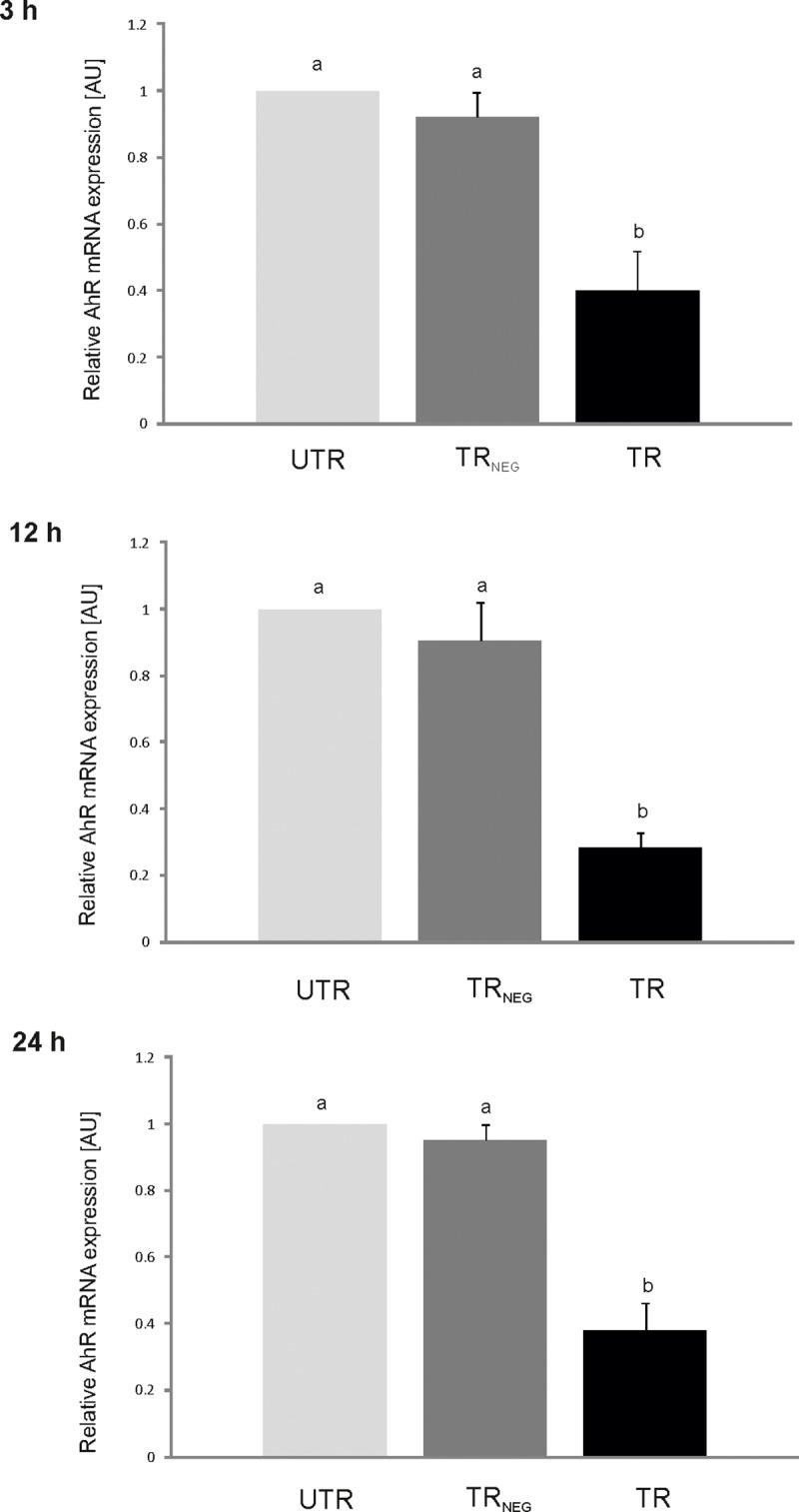
The effects of the *AhR*-targeted siRNA transfection on AhR mRNA expression (mean ± SEM) in porcine granulosa cells. To confirm the silencing of *AhR* gene in porcine granulosa cells, the expression of *AhR* was determined in: 1/ untransfected (UTR) cells, 2/ cells transfected with irrelevant siRNA sequence (TR_NEG_) and 3/ cells transfected with the three relevant siRNAs (TR). After 24 hours of incubation (with pure medium, medium with irrelevant siRNAs or medium with relevant siRNAs), cells were cultured for 3, 12 or 24 h (four biological replicates/time point). AhR mRNA expression level was examined by qRT- PCR and expressed as arbitrary units (AU). mRNA expression level in UTR group was considered as “1”, and the mRNA expression level in TR_NEG_ and TR groups was expressed as a fraction of the UTR value. Stasistical analysis (one-way ANOVA followed by Tukey test) was performed on raw data. Different superscripts designate statistical significant differences (p<0.05) between untransfected UTR, TR_NEG_ and TR groups.

The *AhR*-targeted siRNA transfection significantly reduced the expression level of *AhR* in porcine granulosa cells (Fig 1). The reduction was demonstrated in all time points (3 h, 12 h and 24 h) which were examined to match the experimental design of the following TCDD study. The *AhR* expression level was reduced by 61%, 72% and 63% at 27 (24 h transfection + 3h), 36 (24 h transfection +12 h) and 48 (24 h transfection + 24 h) hours of culture, respectively. The transfection performed with an irrelevant siRNA had no effect on the *AhR* expression level (Fig 1).

### Identification of differentially expressed protein spots in TCDD-treated porcine granulosa cells

**Fig 2:**
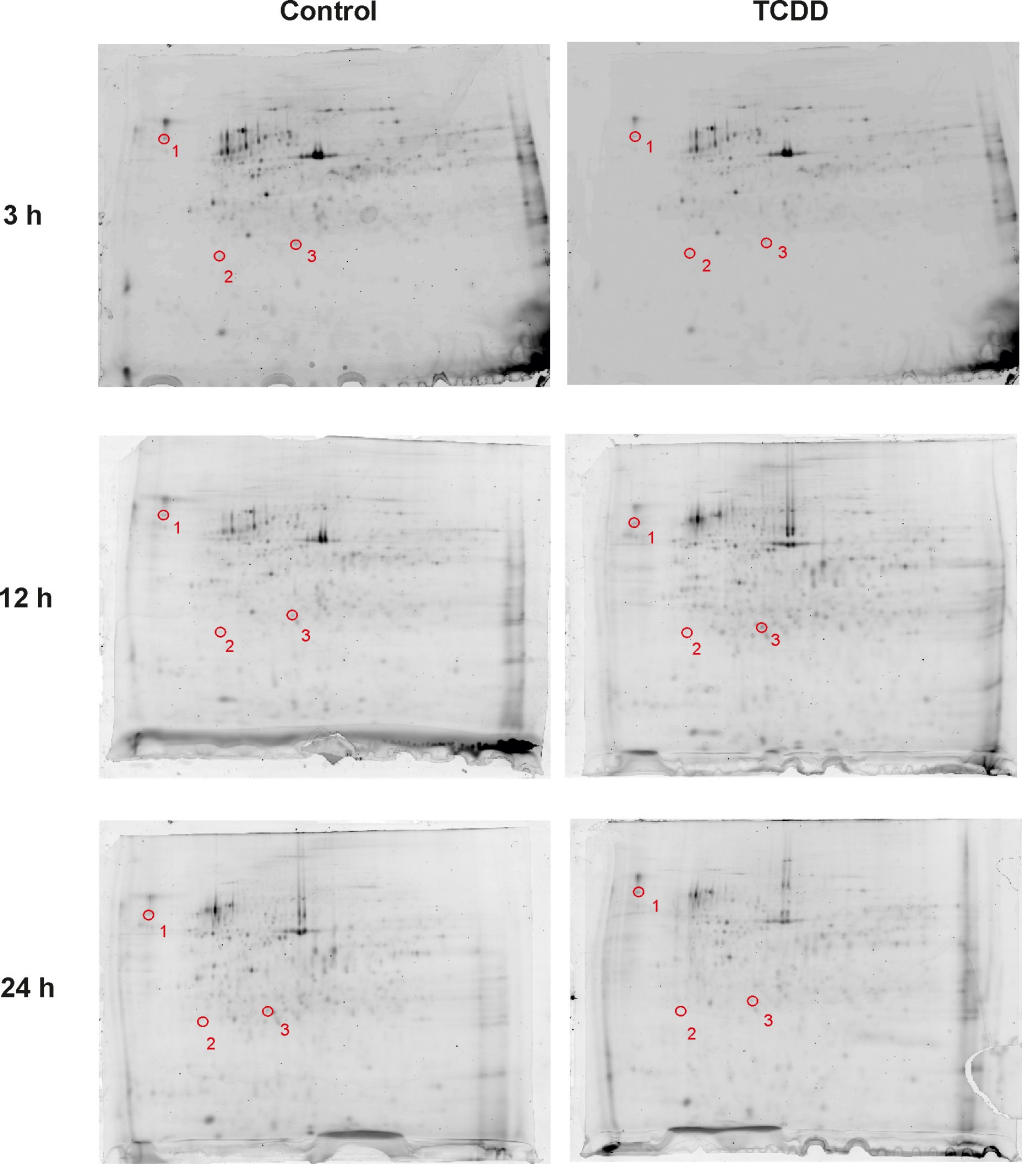
Exemplary images of 2D-DIGE analysis of *AhR*-silenced porcine granulosa cells (TR). The cells were cultured in the absence (Control) and in the presence of TCDD (TCDD) for 3, 12 or 24 h. The numbered protein spots outlined in red represent the proteins that were identified as significantly changed (p<0.05) by TCDD treatment in TR cells.

A DIGE-based proteomic approach was used to identify DEPSs in samples of *AhR*-silenced porcine granulosa cells treated with TCDD for 3, 12 and 24 h. A total of 959 protein spots were detected on all gels and 578 of the protein spots were successfully matched between gels from control (TCDD-untreated cells) and TCDD samples. Within these spots, the abundance of only 3 spots significantly differed (p<0.05 fold change ≥1.5) at each time point between control and TCDD samples. The spots were submitted to MALDI TOF/TOF MS analysis and were identified as AbxA5, PDI and ATPβ. Representative gel images of proteins from control and TCDD-treated cells are presented in Fig 2. The identified proteins are listed and characterized in Table 3.

**Table 3:** The differentially expressed proteins identified in *AhR*-silenced porcine granulosa cells treated with TCDD for 3, 12 and 24 hours.

Spot number	Identified protein	MASCOT protein score	Sequence coverage [%]	Number of peptides (ion score >30)	p-value			Fold change			Accession number
					3 h	12 h	24 h	3 h	12 h	24 h	
1	Annexin V	280	36	3	0.02	0.009	0.02	1.6	1.8	1.5	giǀ 157831404
2	Protein disulfide isomerase	146	19	2	0.006	0.03	0.04	1.9	1.7	1.6	giǀ 1710248
3	Mitochondrial ATP synthase, beta subunit	116	55	3	0.02	0.03	0.02	1.8	1.8	1.7	giǀ 89574037

### Western blotting and immunofluorescence validation of the abundance of selected proteins

**Fig 3:**
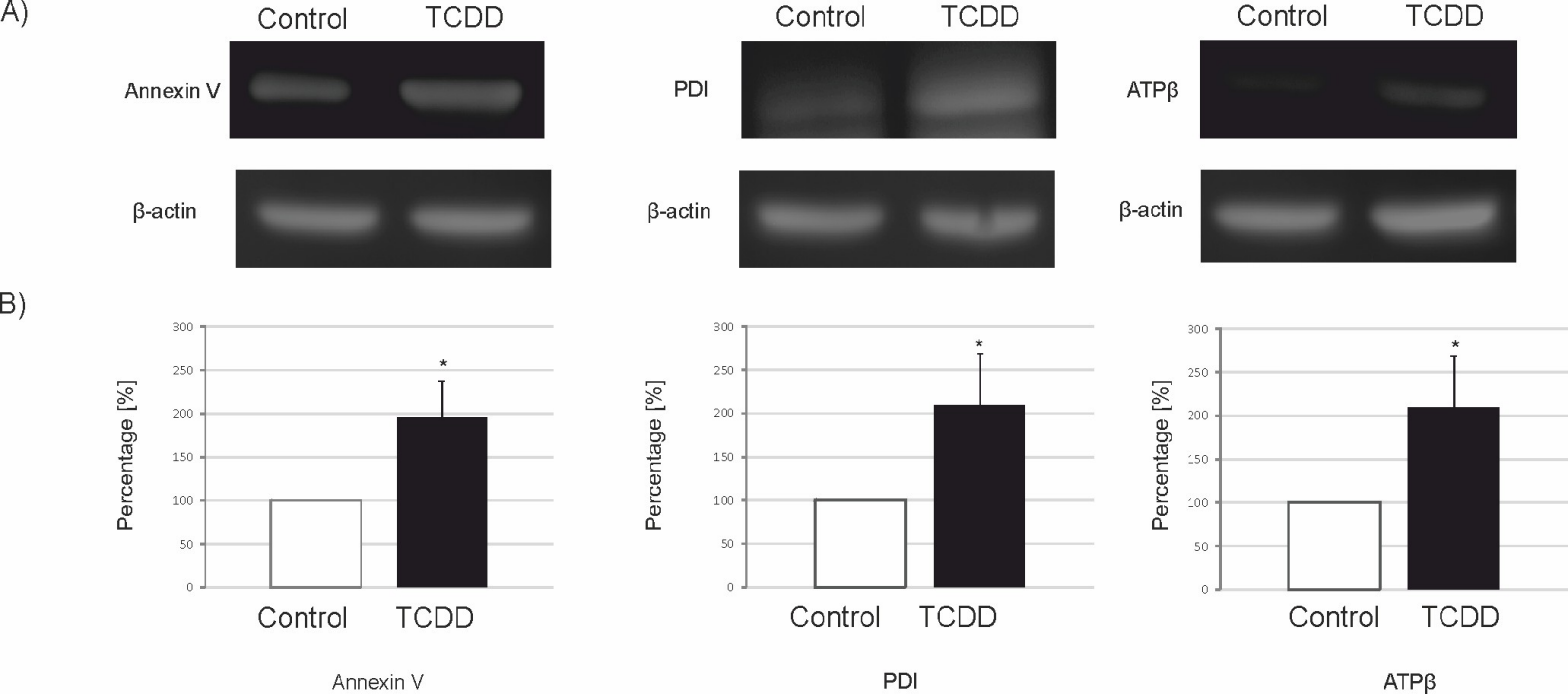
The abundance (mean ± SEM) of annexin V, protein disulfide isomerase (PDI) and mitochondrial ATP synthase, beta subunit (ATPβ) in *AhR*-silenced porcine granulosa cells. The western blotting (WB) validation of 2D-DIGE/ mass spectrometry results was performed in untreated (Control) and TCDD-treated (TCDD) *AhR*-silenced granulosa cells (TR) after 12 h of incubation (n = 3 independent experiments). A) representative immunoblots; B) densitometric analysis of the abundance of the selected proteins performed in relation to β-actin (a reference protein). Data are expressed as a percentage of control samples (100%), * p<0.05.

**Fig 4:**
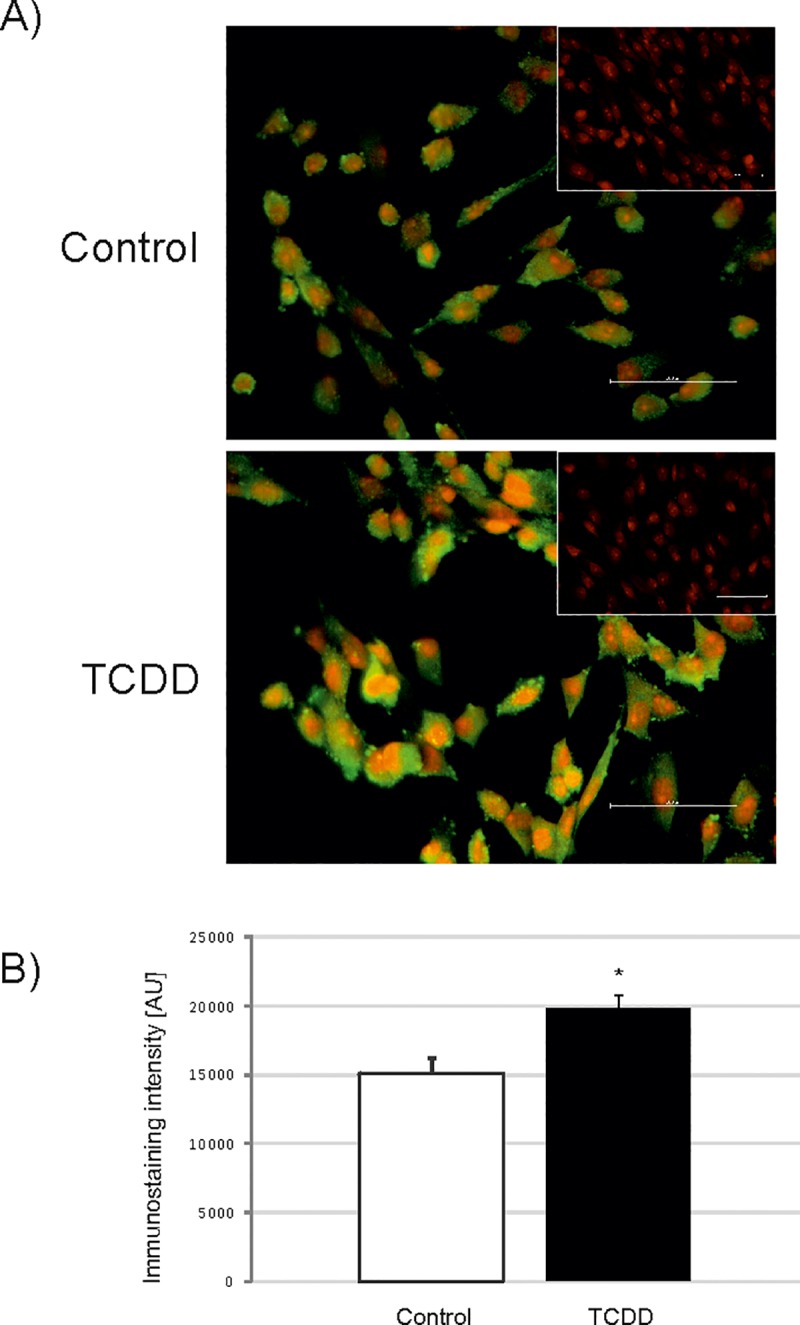
The abundance (mean ± SEM) of annexin V in *AhR*-silenced porcine granulosa cells (TR) determined by immunofluorescence staining. The immunofluorescence validation of 2D-DIGE/ mass spectrometry results was performed in untreated (Control) and TCDD-treated (TCDD) *AhR*-silenced granulosa cells (TR) after 12 h of incubation (n = 3 independent experiments). A) representative images of untreated (Control) cells and cells treated with TCDD (TCDD); green color depicts FITC staining of annexin V and red color depicts propidium iodide staining of the nuclei. The insets represent negative controls, bar = 100 μm; brightness was enhanced for better visualization; B) densitometric analysis of the annexin V abundance; * p<0.05.

**Fig 5:**
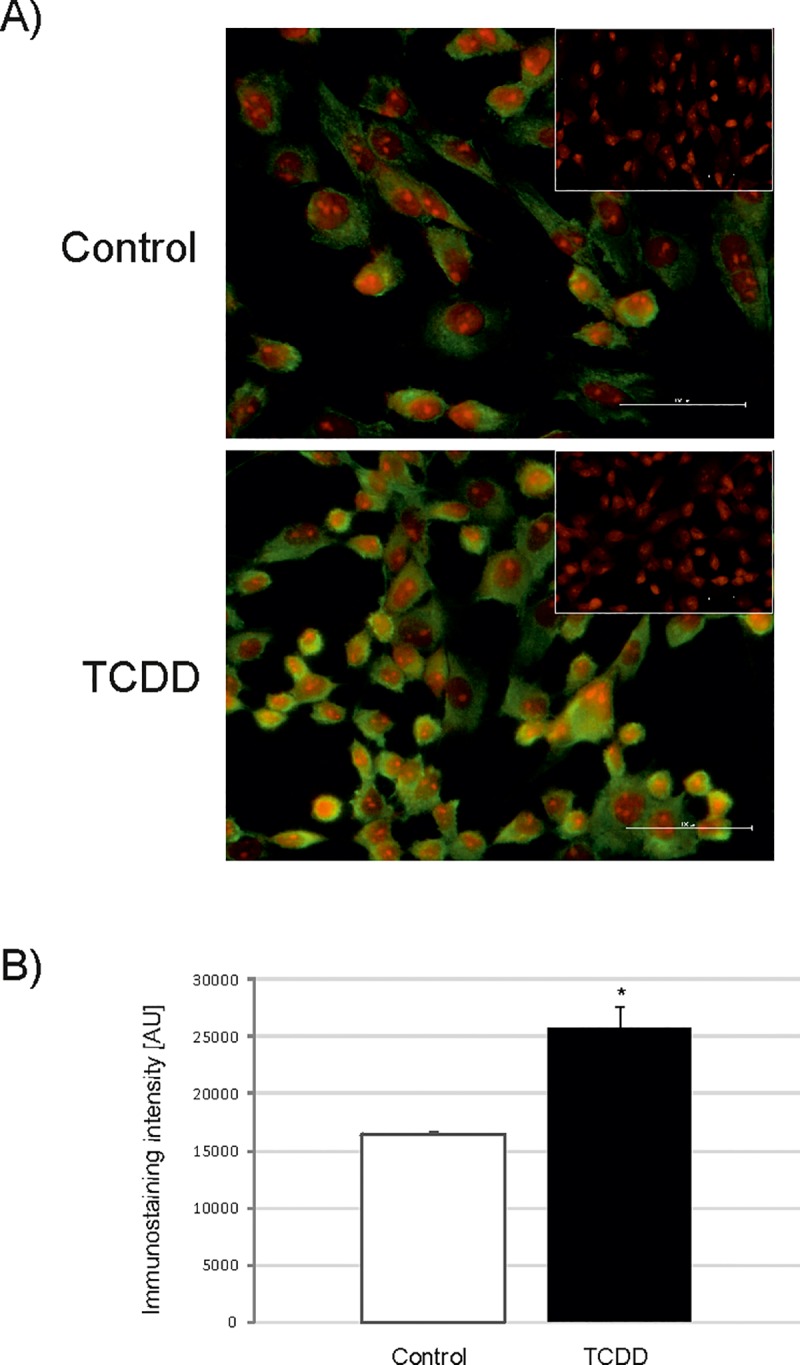
The abundance (mean ± SEM) of protein disulfide isomerase (PDI) in *AhR*-silenced porcine granulosa cells determined by immunofluorescence staining. The immunofluorescence validation of 2D-DIGE/ mass spectrometry results was performed in untreated (Control) and TCDD-treated (TCDD) *AhR*-silenced granulosa cells (TR) after 12 h of incubation (n = 3 independent experiments). A) representative images of untreated (Control) cells and cells treated with TCDD (TCDD); green color depicts FITC staining of PDI and red color depicts propidium iodide staining of the nuclei. The insets represent negative controls, bar = 100 μm; brightness was enhanced for better visualization; (B) densitometric analysis of the PDI abundance; * p<0.05.

The abundance of AnxA5, PDI and ATPβ proteins in porcine granulosa cells (n = 3 independent experiments) was first determined by western blotting. TCDD stimulated (p<0.05) the abundance of all examined proteins (Fig 3). In addition, immunofluorescence was applied to investigate the abundance of AnxA5 and PDI (n = 3 independent experiments). The abundance of these two proteins was upregulated by TCDD (p<0.05), and the presence of both proteins was demonstrated in the cytoplasm of the studied cells (Figs 4 and 5). The obtained results (western blotting and immunofluorescence) confirmed the 2D-DIGE quantification and the accuracy of MS identification for AnxA5, PDI and ATPβ.

## Discussion

A proteomic approach was used in the current study to examine the proteome of *AhR*-silenced porcine granulosa cells exposed to TCDD. Previously, we employed the corresponding intact granulosa cells to study the effects of TCDD on gene [19] and lncRNA [20] expression profiles as well as on the cell proteome [21]. The objective of the present study was to reveal whether TCDD affects the abundance of proteins in the absence of AhR (the main intracellular mediator of TCDD action) and, if yes, to identify proteins associated with the AhR-independent granulosa cell response to the dioxin. To the best of our knowledge, the effects of TCDD on the proteome of *AhR*-silenced ovarian cells were analyzed for the first time.

The *AhR*-targeted siRNA transfection significantly reduced the expression level of *AhR* in porcine granulosa cells in all examined time points. The *AhR* expression level was reduced by 60–70%. The magnitude of the *AhR*-silencing was typical for siRNA transfection method. Due to the resistance of the studied cells to common transfection reagents, such as lipofectamine, our protocol for transfection of AVG-16 cells with siRNA mixture may be a promising tool for cells less susceptible to transfection. Similar to the *AhR* gene expression level, the AhR protein abundance was also significantly decreased after *AhR*-targeted siRNA transfection of the cells using the same transfection protocol (Ruszkowska et al., unpublished). The protein abundance reached 14 ± 3%, 6.7 ± 1.6% and 8.1 ± 2.6% of the level recorded for unsilenced (intact) cells at 3 h, 12 h and 24 h, respectively.

Only three proteins with the abundance significantly affected by TCDD were identified in *AhR*-silenced porcine granulosa cells. In comparison to our recent study pertaining with the effect of TCDD on porcine granulosa cells with a fully active AhR, the magnitude of presently observed changes was rather modest, and ranged from +1.5 to +1.9-fold. In this previous study performed on intact cells, we identified 75 proteins with the abundance significantly affected by the exposure to TCDD [21]. The TCDD-induced proteome changes described in the current study differed from those results. None of the proteins identified after TCDD treatment in intact cells were identified in the *AhR*-silenced granulosa cells. It is safe to assume that TCDD effects in the latter cells may be mediated *via* non-AhR pathway.

Our present data can be discussed only with results obtained from transcriptomic studies, since comparisons of proteomic data between TCDD-treated *AhR*-silenced and TCDD-treated *AhR* intact cells/ organisms are not available. Such transcriptomic studies were conducted on mice [29, 30]. It was demonstrated that TCDD administration affected the hepatic expression of only 32 genes in AhR-null mice in comparison to 297 genes in wild type animals (WT) [29]. Similarly, it was reported that the exposure of AhR-null mice to TCDD produced significant changes in the kidney expression of only 5 genes compared to 17 genes with TCDD-altered expression identified in WT mice [30]. The considerably lower number of genes or proteins with the expression or abundance significantly affected by TCDD in the absence of AhR, supports the notion that Ah receptor is the main mediator of TCDD action. Nevertheless, the fact that TCDD was able to induce changes in the expression of some genes or in the abundance of some proteins in the *AhR*-silenced environment, suggests that some AhR-independent pathways are also involved in TCDD signaling.

In the present study, TCDD increased the abundance of PDI, ATPβ and AnxA5 in the *AhR*-silenced porcine granulosa cells. PDI resides usually in the endoplasmic reticulum (ER), but it can be also found in the nucleus and cytoplasm [31]. PDI functions involve reduction and isomerization of disulfide bonds as well as oxidation of thiols, it also demonstrates chaperone activity. Therefore, PDI plays an essential role in protein folding and quality control of proteins, providing their proper structural stability and shaping the active sites of enzymes [32]. Previously, we demonstrated that TCDD increased the abundance of hsp70 [21] in intact porcine granulosa cells. Hsp70 is responsible for protein folding as well as for the repair and degradation of damaged or misfolded proteins. The TCDD-induced upregulation of PDI and hsp70 in *AhR*-silenced and intact granulosa cells, respectively, indicates that TCDD, regardless of the AhR status of the cell, may activate similar processes although by employing different signaling molecules.

It should be also emphasized that PDI is a major calcium (Ca^2+^) binding protein of the ER. Due to its high capacity to bind Ca^2+^ with low affinity, PDI regulates intracellular calcium homeostatis [33, 34]. The optimal intracellular Ca^2+^ concentration is necessary for protein folding as well as it facilitates protein degradation [35]. TCDD and other polycyclic aromatic hydrocarbons (PAHs) such as benzo(a)pyrene [B(a)P] were reported to increase, in an AhR-independent manner, the intracellular concentration of Ca^2+^ [36, 37]. It is possible that the elevated abundance of PDI may protect cells against TCDD-mediated disruption of calcium homeostasis.

ATPβ, abundance of which was also enhanced by TCDD in the *AhR*-silenced cells, is a catalytic subunit of ATP synthase F_1_ region. ATP synthase produces ATP, the energy storage molecule. TCDD was demonstrated to decrease ATP level in mice liver [38] and JAR cells (cell derived from a human trophoblastic tumor of the placenta) [39], both possessing a fully active AhR gene. TCDD also increased the incidence of apoptosis in the latter cells [39]. The role of ATP and ATP synthase in apoptosis has been intensively investigated. It was reported that the specific inhibitor of ATP synthase induced apoptosis in numerous cells [40, 41, 42, 43]. On the other hand, a high expression level of catalytic subunit of ATP synthase (ATP5A1) was reported in proliferating cancer cells [44]. These findings suggest that the TCDD-increased abundance of ATPβ in the *AhR*-silenced porcine granulosa cells may be associated with anti-apoptotic effects of the dioxin.

The last identified protein with the abundance increased by TCDD in the *AhR*-silenced porcine granulosa cells was AnxA5. Annexins are phospholipid-binding proteins classified into five groups (A–E), with 12 members found in vertebrates (AnxA1–A13, AnxA12 is unassigned) [45, 46]. These proteins bind to phospholipids in a Ca^2+^-dependent manner. AnxA5 is the most abundant member of the annexin family and is expressed in most cells and tissues except neurons [46, 47]. AnxA5 is associated with membrane trafficking and organization, Ca^2+^ signaling, regulation of ion channels and Ca^2+^-influx as well as cell cycle regulation and apoptosis [46, 48]. Despite the fact that the role of AnxA5 in many processes was intensively investigated, the function of this protein in apoptosis remains unclear. On one hand, the cell viability was significantly increased in *AnxA5*-silenced human renal epithelial cells (HK-2 cell line) in comparison to untransfected cells, suggesting that AnxA5 is involved in processes leading to cell death [49]. This is consistent with the fact that AnxA5 overexpression increased the activity of caspase-3 in murine cardiomiocytes and calf chondrocytes [50, 51]. On the other hand, AnxA5 counteracted apoptosis induced by etoposide (apoptosis inducing factor) and delayed the activation of caspase-3 in human CEM T-lymphoma cells [52]. Moreover, in contrast to mouse perivascular and human trophoblast cells lacking AnxA5, the corresponding wild type cells were able to repair damaged membranes [53, 54]. However, we were not able to demonstrate that 100 pM-100 nM of TCDD affect the incidence of apoptosis in porcine granulosa cells harvested from medium and preovulatory porcine follicles after 48 hours of culture [21, 55]. Nevertheless, the TCDD-affected abundance of AnxA5 suggests that the role of AnxA5, not necessarily in apoptosis, should be further explored in granulosa cells by employing functional studies.

## Conclusions

The obtained results revealed the ability of TCDD to alter protein abundance in an AhR-independent manner although TCDD influenced the abundance of only three proteins. These proteins were identified as PDI, ATPβ and AnxA5 and were demonstrated to be involved in Ca^2+^ signaling, membrane trafficking and organization, protein folding, energy storage as well as cell cycle regulation and apoptosis. The present study offers a broader insight into the mechanism of TCDD action and provide new directions for future functional studies focused on molecular effects exerted by TCDD.

## Supporting information

S1 Table Labeling scheme of the control and TCDD-treated samples* examined by 2D-DIGE. *the samples originated from *AhR*-silenced porcine granulosa cells (TR)–untreated and treated with TCDD (100 nM) **a mixture of each experimental sample listed in columns 3 and 4 of the table.
(DOC)

S2 Table List of the mass over charge (m/z) of precursor ions and the sequence of each identified peptide. (DOC)
