# Supplementary material for: Proteomic changes of aryl hydrocarbon receptor (AhR)-silenced porcine granulosa cells exposed to 2,3,7,8-tetrachlorodibenzo-p-dioxin (TCDD)
Source: PLoS One. 2019 Oct 4;14(10):e0223420. doi: 10.1371/journal.pone.0223420 (PMC6777791; doi:10.1371/journal.pone.0223420)
Supplement: S1 Table — *the samples originated from AhR-silenced porcine granulosa cells (TR)–untreated and treated with TCDD (100 nM) **a mixture of each experimental sample listed in columns 3 and 4 of the table. (DOC) [file pone.0223420.s001.doc]

S1 Table. Labeling scheme of the control and TCDD-treated samples* examined by 2D-DIGE

| Gel number | Cy2 | Cy3 | Cy5 |
| --- | --- | --- | --- |
| 1 | internal standard** | control 3 h (a) | TCDD 3 h (a) |
| 2 | internal standard | control 3 h (b) | TCDD 3 h (b) |
| 3 | internal standard | TCDD 3 h (c) | control 3 h (c) |
| 4 | internal standard | TCDD 3 h (d) | control 3 h (d) |
| 5 | internal standard | control 12 h (a) | TCDD 12 h (a) |
| 6 | internal standard | control 12 h (b) | TCDD 12 h (b) |
| 7 | internal standard | TCDD 12 h (c) | control 12 h (c) |
| 8 | internal standard | TCDD 12 h (d) | control 12 h (d) |
| 9 | internal standard | control 24 h (a) | TCDD 24 h (a) |
| 10 | internal standard | control 24 h (b) | TCDD 24 h (b) |
| 11 | internal standard | TCDD 24 h (c) | control 24 h (c) |
| 12 | internal standard | TCDD 24 h (d) | control 24 h (d) |

*the samples originated from *AhR*-silenced porcine granulosa cells (TR) – untreated and treated with TCDD (100 nM)

**a mixture of each experimental sample listed in columns 3 and 4 of the table

a,b,c,d – depict the subsequent four biological replicates
