# Supplementary material for: Proteomic changes of aryl hydrocarbon receptor (AhR)-silenced porcine granulosa cells exposed to 2,3,7,8-tetrachlorodibenzo-p-dioxin (TCDD)
Source: PLoS One. 2019 Oct 4;14(10):e0223420. doi: 10.1371/journal.pone.0223420 (PMC6777791; doi:10.1371/journal.pone.0223420)
Supplement: S2 Table — (DOC) [file pone.0223420.s002.doc]

S2 Table. List of the mass over charge (m/z) of precursor ions and the sequence of each identified peptide

| Spot number in Figure 2 | Protein name | Mass over charge (m/z) of precursor ions | Sequence of identified peptides |
| --- | --- | --- | --- |
| 1 | annexin V | 1340.6995  1704.9814  2888.3177  1172.7486  1818.9261  1613.9719  1106.6651  1834.9384 | R.GTVTDFPGFDER.A  K.GLGTDEESILTLLTSR.S  K.YMTISGFQIEETIDR.E  R.SEIDLFNIR.K |
| 2 | protein disulfide isomerase | 2057.0737  1191.6139  1386.8283  1527.9080  1151.5706  1483.8030 | -.LYSSSDDVIELTPSNFNR.E  K.NRPEDYQGGR.T  K.LAAVDATVNQVLASR.Y  K.GSFSEQGINEFLR.E |
| 3 | mitochondrial ATP synthase, beta subunit | 1651.2589  1278.9029  1038.8383  1401.9816  1458.0410  1618.1223  1440.0992  1922.3503  1436.0604  2298.5442  1988.4259  3032.1395  2691.7653  2564.6133 | K.IPVGPETLGR.I  R.VALTGLTVAEYFR.D  R.DQEGQDVLLFIDNIFR.F |
